# Evaluation of computed tomography artefacts of carbon-fiber and titanium implants in patients with spinal oligometastatic disease undergoing stereotactic ablative radiotherapy

**DOI:** 10.1038/s41598-024-52498-2

**Published:** 2024-03-20

**Authors:** Zeger Rijs, Khandkar Ali Kawsar, Priyanshu Saha, Michiel van de Sande, Darren Lui

**Affiliations:** 1 Department of Orthopedic Surgery, Leiden University Medical Center, Leiden, The Netherlands; 2 Department of Orthopedic and Spinal Surgery, St. George’s Hospital, London, UK; 3 Department of Neurosurgery, Royal Infirmary of Edinburgh, Edinburgh, UK

**Keywords:** Surgical oncology, Bone cancer

## Abstract

This study evaluated artefacts on computed tomography (CT) images using Hounsfield units (HU) in patients with spinal oligometastatic disease who received carbon-fiber (CF; n = 11) or titanium (n = 11) spine implants and underwent stereotactic ablative radiotherapy (SABR). Pre- and postoperative HU were measured at the vertebral body, pedicle, and spinal cord at three different levels: the lower instrumented vertebra, the level of metastatic spinal cord compression, and an uninvolved level. Areas measured at each level were delicately matched pre- and postoperatively. Significant differences in HU were observed at the vertebral body, the pedicle, and the spinal cord at the lowest instrumented vertebra level for both CF and titanium (average increase 1.54-fold and 5.11-fold respectively). At the metastatic spinal cord compression level, a trend towards a higher HU-increase was observed in titanium compared with CF treated patients (average increase 2.51-fold and 1.43-fold respectively). The relatively high postoperative HU-increase after insertion of titanium implants indicated CT artefacts, while the relatively low HU-increase of CF implants was not associated with artefacts. Less CT artefacts could facilitate an easier contouring phase in radiotherapy planning. In addition, we propose a CT artefact grading system based on postoperative HU-increase. This system could serve as a valuable tool in future research to assess if less CT artefacts lead to time savings during radiotherapy treatment planning and, potentially, to better tumoricidal effects and less adverse effects if particle therapy would be administered.

## Introduction

Spinal metastases are common in oncological care as approximately 70% of all bony metastasized cancers are located in the spine^1,2^. Spinal oligometastatic disease (OMD) is defined as a subgroup of patients with limited (≤ 5) metastatic lesions in the spine where all metastatic sites are safely treatable^3,4^. Treatment of spinal OMD is a multidisciplinary team effort, and management must be individualized for each patient. Factors that impact treatment strategy include histology, tumor location, symptoms, radiosensitivity, and prior treatment^5^. Surgery can be performed in case of mechanical pain, decompression, correction of instability or deformity, and with the purpose of oncological cytoreduction^6^. In patients with limited spinal OMD, surgery combined with postoperative radiotherapy (RT) to improve local control is an established practice^7^.

Conventional external beam radiation therapy (EBRT) to the entire spine has been the golden standard for decades due to its excellent palliative effect. However, EBRT doses are too low to ensure long term local control, and raising the dose is not an option because the spinal cord is often at risk^8^. Fortunately, stereotactic ablative radiotherapy (SABR) is an emerging noninvasive approach for the treatment of spinal OMD^9^. It has drastically changed the treatment from palliative to curative care for several (early detected) cancers, including lung-, liver-, prostate-, breast-, and spine cancer^10–13^. SABR can precisely deliver tumoricidal radiation doses to the tumor(s), while sparing adjacent tissues, thereby achieving durable local tumor control with low complication rates^10^. This is a delicate procedure that highly depends on accuracy, not only because the dose must be high enough to be toxic to tumor cells, but also because it requires high precision as the spinal cord is often right next to the area being treated^14^. Therefore, precise SABR planning with computed tomography (CT), or magnetic resonance imaging (MRI), is essential to ensure optimal treatment for spinal OMD^15^. Several prospective trials have already demonstrated that SABR is an effective tool for treating spinal OMD^16–20^.

Although very promising, a major challenge in the delivery of SABR to spinal OMD is the proximity of the spinal cord. Despite technical evolutions such as surface-guided monitoring systems, metal artefact reduction, and couch corrections in all six degrees of freedom, SABR treatment can be hampered when spinal tumors are treated with titanium (or other metallic) implants^21^. Commonly used titanium implant materials produce substantial artefacts on CT images^22^. Consequently, these implants pose problems with respect to (time-consuming) radiation planning and accurate delivery of the calculated dose^22^. This could lead to complications such as spinal cord radio necrosis, progressive myelopathy, spinal hemorrhage, and fractures^23,24^. Tedesco et al. reported that scattering of radiotherapy from titanium spine implants can compromise the therapeutic effect and lead to unwanted radiation to adjacent healthy tissue^25^. In addition, titanium (or other metallic) artefacts also interfere with postoperative radiologic surveillance used to track bone healing and identify recurrences^26^.

A possible improvement for spinal OMD treatment with SABR is to change traditional titanium (or other metallic) implants to innovative carbon-fiber (CF) implants. CF materials have good biocompatibility, chemical stability, good mechanical properties, and a modulus of elasticity which is similar to human bone and theoretically leads to better bone quality^27^. Besides, clinical studies have not shown an increase in complications with implementation of CF implants^25,28–30^. Therefore, CF implants could improve SABR planning and lead to more accurate delivery of the calculated dose compared to traditional implants^31^. Several CF spine implants have shown promising results with regards to reducing artefacts, better radiation planning, and potentially greater safety and quality of radiotherapy^25,32,33^. However, it has been difficult to quantify the difference in CT artefacts after implementation of CF and titanium implants. In the current study, we utilized a quantitative technique to perform a pre- and postoperative comparison of CT artifacts produced by CF and titanium implants in patients with spinal OMD undergoing postoperative SABR. In addition, we propose an artefact grading system to classify CT artefacts.

## Materials and methods

This retrospective single center study included patients ≥ 18 years with spinal OMD who received CF or titanium spinal implants (including pedicles, screws, and rods) between 2018 and 2020. A closely matched gender and age group of patients that received CF and titanium implants was selected because gender- and age-related osteoporotic changes in bone density could potentially influence CT artefact measurements. Patients with traumatic or inflammatory conditions, or previous fusion surgery were not eligible. Additionally, patients that received bone cement (i.e., polymethyl methacrylate or PMMA) at the level of metastasis or instrumented level were excluded because this could potentially interfere with CT artefact measurements. The study protocol was approved by the local ethics committee (St. George’s Research Ethics Committee, clinical audit registration number AUDI003026), and informed consent was obtained from all subjects and/or their legal guardians. All methods were performed in accordance with relevant guidelines and regulations.

Surgery was recommended as a curative treatment strategy in patients with spinal OMD. Most patients presented with pain, some with spinal cord compression, and response to non-surgical treatment was insufficient. Decompression and fixation surgery was predominantly performed for those cases, and there was no standardized protocol to choose for CF implants instead of titanium implants. Therefore, the choice between CF or titanium was made by shared decision making and the preference of the operating surgeon. During this study, patients were treated with various FDA approved and CE marked CF (CarboFix Orthopedics; Herzliya, Israel) and titanium implants (Stryker Corporation, Michigan, United States of America).

### Outcomes assessment

**Figure 1:**
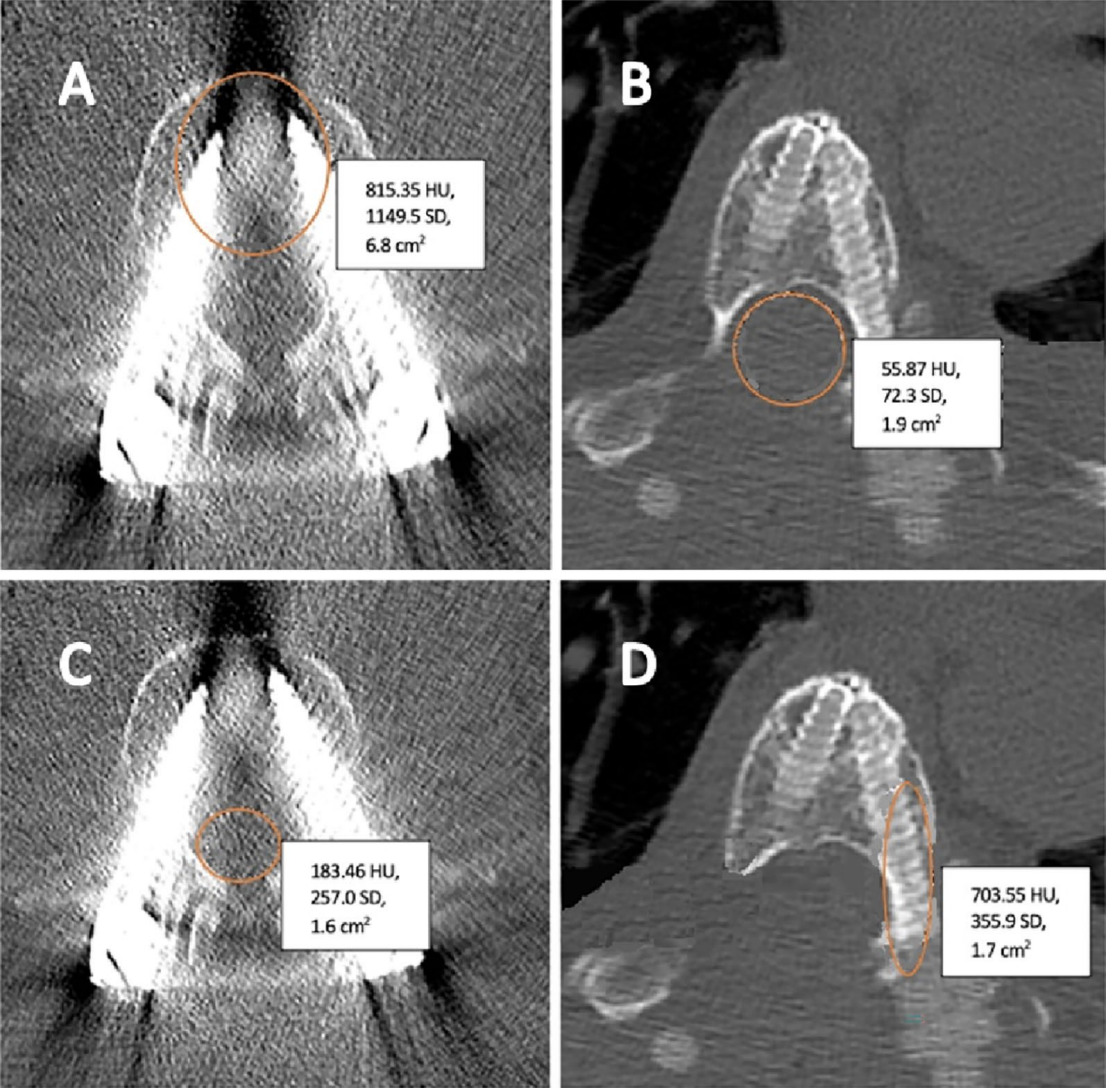
Hounsfield unit measurement of the vertebral body of the lower instrumented vertebra with bright and dark artefacts after treatment with a titanium implant (**A**), measurements of the spinal cord near the lower instrumented vertebra with carbon-fiber (**B**), measurements of the spinal cord near the lower instrumented vertebra with titanium (**C**), and an elliptical measurement of the pedicle of the lower instrumented vertebra with carbon-fiber without bright or dark artefacts (**D**).

Artefacts on CT images were measured preoperatively and within the first postoperative week using Hounsfield units (HU), which were determined by a picture archiving and communication system (PACS) integrated software (Phillips Medical Systems, Eindhoven, The Netherlands). An unmodified standard care CT spine protocol (median tube potential 140kVp, median tube current 60mA, 2.5mm slice thickness) was used to measure the artefacts; the software measured the density of a region of interest (ROI), which electronically overlaid the image, and calculated the corresponding HU. ROI were selected by a neurosurgeon (K.A.K.) and checked by an orthopedic surgeon specialized in spine surgery (D.L.). Pre- and postoperative HU measurements were performed at the vertebral body, pedicle, and spinal cord at three different levels: the lower instrumented vertebra, the level of metastatic spinal cord compression, and at an uninvolved level. These locations were chosen because the lower instrumented vertebra received the implant. The level of metastatic spinal cord compression was chosen because this is the level where radiotherapy is directed, and usually no screws are inserted at this level which means that HU changes come from scattering from different levels. Additionally, an uninvolved level served as internal control. Areas were measured as a circle in the vertebral body and spinal canal, while an ellipse was used for the pedicle (Fig. 1). CT images were analyzed for the CF as well as the titanium implant group, and HU areas measured at each level were delicately matched between pre- and postoperative images (< 10% difference in the examined area measured in cm^2^). In addition, a CT artefact grading system was developed based on the postoperative HU change.

### Statistical analysis

Statistical analyses were performed using SPSS version 25 (IBM Corp, Somer, NY, USA). For continuous data, the Kolmogorov–Smirnov test was used to assess the normal distribution assumption. Mean HU were calculated for each group pre- and postoperatively. These mean HU values were used because the distribution of our data was symmetric without clear outliers, and a paired t-test was used to compare the mean HU pre- versus postoperatively. Significance was set as a *p*-value ≤ 0.05.

## Results

In total, 22 patients were included. Six males and five females (n = 11) were included in the CF implant group, with a mean age of 54 years (range 20–70 years). The titanium group consisted of five males and six females (n = 11), with a mean age of 56 years (range 36–66 years). Although two patients in the CF group were primary spine tumors, most of the lesions were spinal metastasis, and the involved location was mostly at the thoracic level of the spine (Table 1).

**Table 1:** Demographic features of included patients treated with carbon-fiber or titanium implants.

Baseline characteristics	Carbon-fiber implant group (n = 11)	Titanium implant group (n = 11)
Female % (n of total)	45% (n = 5)	55% (n = 6)
Age (years; mean with range)	54 (20–70)	56 (36–66)
Primary tumor		
Primary spine tumor	18% (n = 2)	0% (n = 0)
Metastasis*	82% (n = 9)	100% (n = 11)
Level of the lesion		
Cervical	9% (n = 1)	9% (n = 1)
Thoracic	64% (n = 7)	55% (n = 6)
Lumbar	27% (n = 3)	36% (n = 4)

* Metastasis most frequently originated from renal cell carcinoma (n = 3) and lung cancer (n = 3), followed by breast-, prostate-, and thyroid cancer (n = 2 per group) and a group of other types of cancer including bladder cancer, gastric cancer, melanoma, ovarian cancer, plasmacytoma, and sarcoma (n = 1 per type of cancer).

### Pre-versus postoperative HU comparison for carbon fiber implants

The average HU of the vertebral body, pedicle, and spinal cord at the level of the lower instrumented vertebra, at the metastatic spinal cord compression level, and at the uninvolved level was compared before and after insertion of the CF implants. CF instrument scatter artefacts were observed with higher postoperative HU. Although HU only increased with a maximum of 1.67-fold compared to its preoperative value, there was a significant increase at the vertebral body-, pedicle-, and spinal cord at the lowest instrumented vertebra level (*p* = 0.012, 0.015, and 0.014 respectively; Table 2). No statistically significant HU-increase was observed at the metastatic spinal cord compression level (generally no instrumentation at the tumor level) and at the uninvolved level (internal control) (Table 2).

**Table 2:** Hounsfield unit comparison within the carbon-fiber group.

Level	Preoperative HU*	Postoperative HU*	fold increase	p-value
LIV VB	163.19	267.67	1.64	**.012**
LIV ped	298.20	493.88	1.65	**.015**
LIV SC	36.27	47.81	1.32	**.014**
MSCC VB	236.46	394.63	1.67	.089
MSCC Ped	230.05	367.93	1.60	.910
MSCC SC	49.92	50.82	1.02	.125
Uninvolved level VB	137.26	141.77	1.03	.667
Uninvolved level Ped	282.86	286.37	1.01	.376
Uninvolved level SC	30.11	34.57	1.15	.261

LIV, Lowest Instrumented Vertebra; VB, Vertebral Body; SC, Spinal Cord; Ped, Pedicle; MSCC, metastatic spinal cord compression; HU, Hounsfield unit.

*Average values of all included patients are reported.

Significant are in value [bold].

### Pre-versus postoperative HU comparison for titanium implants

The average HU of the vertebral body, pedicle, and spinal cord at the level of the lower instrumented vertebra, at the metastatic spinal cord compression level, and at the uninvolved level was compared before and after insertion of the titanium implants. Titanium instrument scatter artefacts were observed with higher postoperative HU. The same trend was observed as with CF implants, with significantly increased HU postoperatively (maximum 5.65-fold increase) at the vertebral body-, pedicle-, and spinal cord at the lowest instrumented vertebra level (*p*-values of 0.00, 0.00, and 0.24 respectively; Table 3). Although not statistically significant, a trend towards higher HU was observed in titanium implants (average 2.51-fold HU-increase) at the metastatic spinal cord compression level. As expected, no significant HU-increase was observed at the uninvolved level (internal control).

**Table 3:** Hounsfield unit comparison within the titanium group.

Level	Preoperative HU*	Postoperative HU*	x increase	*p*-value
LIV VB	166.69	790.28	4.74	**.000**
LIV ped	249.49	1386.78	5.56	**.000**
LIV SC	29.37	147.49	5.02	**.024**
MSCC VB	174.39	530.01	3.04	.155
MSCC Ped	263.47	419.09	1.59	.058
MSCC SC	35.91	103.92	2.89	.243
Uninvolved level VB	195.02	177.18	0.91	.151
Uninvolved level Ped	295.26	283.86	0.96	.053
Uninvolved level SC	30.35	38.05	1.25	.082

LIV, Lowest Instrumented Vertebra; VB, Vertebral Body; SC, Spinal Cord; Ped, Pedicle; MSCC, metastatic spinal cord compression; HU, Hounsfield unit.

*Average values of all included ptients are reported.

Significant are in value [bold].

### Artefact grading system to classify CT artefacts

Based on our observations the postoperative HU-increase was associated with an increase in CT artefacts. Therefore, we propose a CT artefact grading system where grade 0 = no increase (i.e., bone allografts), grade 1 = 1–1.3-fold (differences in study planes), grade 2 = 1.3–2-fold increase (CF), grade 3 = 2–4-fold increase (i.e., CF with cement), and 5 =  > 4-fold increase (titanium) (Table 4). This grading system provides information on the ability to assess anatomically relevant structures and could be used in future studies to assess if less CT artefacts indeed facilitate an easier contouring phase in radiotherapy planning and possibly lead to better tumoricidal effects and decreased adverse outcomes in cases where newer forms of radiotherapy, such as particle therapy, would be considered.

**Table 4:** Computed tomography artefact grading system based on postoperative Hounsfield unit increase.

Grade	Fold increase of HU	Description, assessment of anatomically relevant structures
Grade 0:	Less than 1	Bone allografts, perfect assessment
Grade 1:	1–1.3	Differences in study planes, very good assessment
Grade 2:	1.3–2	Carbon fiber implants, good assessment
Grade 3:	2–4	Cement was observed to increase HU, moderate assessment
Grade 4:	4 and above	Titanium implants, poor assessment

## Discussion

In this study, we utilized a quantitative technique to perform a pre- versus postoperative comparison of HU produced by CF and titanium implants in patients with spinal OMD undergoing postoperative SABR. Significant increases in HU were observed at the vertebral body, the pedicle, and the spinal cord at the lowest instrumented vertebra level for both CF and titanium implants (average 1.54-fold and 5.11-fold HU-increase, respectively). At the metastatic spinal cord compression level, a trend towards a higher HU-increase was observed in titanium implants compared with CF implants (average 2.51-fold and 1.43-fold HU-increase respectively). In general, no screws are inserted at this level, which means the HU-increase comes from the scatter of a cage or rods posteriorly. As expected, no postoperative HU-increase was observed at the uninvolved level for both CF and titanium. Based on our observations, the HU-increase indicates an increase in CT artefacts. Therefore, we propose a CT artefact grading system based on postoperative HU-increase, which provides information on the ability to assess anatomically relevant structures and could be used in future long term follow up studies. These studies could assess if less CT artefacts (low grade artefacts) indeed lead to time savings during radiotherapy planning and, potentially, to enhanced tumoricidal effects with less adverse outcomes in cases where particle therapy would be administered.

A recent in vitro study of Krätzig et al. evaluated the susceptibility of artefacts in CT and MRI of titanium and CF screw-rod constructs for posterior spinal stabilization using a standardized in vitro model^34^. Here, similar manually placed 2D ROI were defined for each image, and CT imaging with typical implant configuration for thoracic stabilization demonstrated a significant artifact reduction in CF compared with titanium implants for the evaluation of index structures, such as the spinal cord and the vertebra. Coherently, Depauw et al. used a water phantom as a human tissue equivalent and reported no imaging artefacts and minimal dose perturbation of CF compared with titanium^35^. In addition, Fleege et al. reported reduced artifacts of CF pedicle screws in MRI scans of patients with lumbar spondylodesis^36^. The authors calculated the surface of the artifact free vertebral body area as percentage of the total vertebral body, and CF displayed significantly less artefacts than titanium (67.1 ± 5.6% vs. 48.3 ± 5.0%; *p* ≤ *0.01*, respectively). Furthermore, Ringel et al. reported reduced artefacts of CF spine implants compared with titanium implants and conclude that CF spine implants are a valuable and feasible option in spine tumors where postoperative imaging and radiation planning are necessary^26^. Our findings, together with the previously mentioned studies, highlight that CF spine implants show reduced artefacts compared to titanium.

The clinical relevance of the reduced artefacts after CF implementation instead of titanium remains to be further elucidated. New treatment planning systems, which outline metallic materials and associate an atomic number which is used for dose calculation purposes (the density override method), correct for metallic artefacts. Therefore, metallic implants are unlikely to impact the tumoricidal effects of SABR. However, the density override method no longer needs to be applied with CF implants, which results in a simpler method and therefore time savings, as well as an accurate dose distribution^37^. Besides, radiation oncologists are increasingly interested in CF spinal instrumentation because it enables the use of particle therapy, such as proton beam therapy, in a group of patients where it was previously impossible due to the imaging artefacts and perturbation effect of metallic instrumentation^38^. Several studies have shown that the use of CF is favorable to titanium instrumentation for the use in particle therapy. Nevelsky et al. investigated the perturbation effect of CF screws compared to titanium screws and found a perturbation effect of less than 5% for CF screws, compared to greater than 30% for titanium screws^39^. Mastella et al. evaluated the dosimeric perturbation caused by CF screws compared to titanium screws and found less dose degradation caused by CF screws, making CF more suitable for particle therapy^40^. Ultimately, this might help achieving the goal of durable tumor control with low complication rates.

Drawbacks of CF implants include its potential challenging surgery due to its radiolucency. However, recent research has shown CT-guided navigation of pedicle screws is possible for instrumentation and precision assessment across the thoraco-lumbar spine^41^. Besides, long-term postoperative results of CF implants, including the effect of radiation on the properties of CF implants, remain to be investigated. Some also question whether availability and costs of CF implants could be a disadvantage. Although we are not aware of its availability, production costs have decreased as CF composites are widely used across other industries, and current costs of CF nails are competitive with conventional metal nails^42^.

This study has several limitations. First, our objective quantitative assessment using HU is not a perfect measurement of artefacts. Although several studies have used HU to predict osteoporosis and artefacts, we acknowledge that CT scanner configurations, depth of the measurement, location of the measurement, tissue type, implant material and artefacts due to motion during the scan can all influence HU^34,43^. Artefacts can be bright (high HU) or dark (low HU). Therefore, postoperative HU differences, such as our CT artefact grading system based on postoperative HU-increase, might only be a surrogate marker for CT artefacts. Nevertheless, this real life setting with standard clinical protocols provides relevant clinical insights; the relatively high HU-increase after insertion of titanium implants indicated CT artefacts, while the relatively low HU-increase after insertion of CF implants was not associated with artefacts. A qualitative assessment by a musculoskeletal radiologist could contribute to the validity and reliability of our study because this is generally seen as the ground-truth. Although not performed in this study, a qualitative analysis of post-operative artifact-free vertebrae surface area and its ratio to pre-operative vertebrae surface area, as done by Fleege et al., would presumably show better results in patients treated with CF implants^36^. Another limitation is that our proposed CT artefacts grading system is solely based on 11 patients receiving CF implants and 11 patients receiving titanium implants. Validation is needed to assess if this HU based classification system holds promise for assessing CT artefacts in future studies. Furthermore, our retrospective study design is inherently susceptible to several forms of bias, including selection- and assessor bias, and causal differences should be interpreted with caution. However, we objectively measured carefully matched ROI and compared the hardware against itself (in different levels) and against its own control (pre- and postoperatively). Postoperative HU differences which are clinically relevant for the ability to assess anatomically relevant structures or metastasis/residual tumor, improve planning (time savings) and accurate administration of newer forms of radiotherapy, such as particle therapy, should be further examined.

## Conclusion

It has been difficult to quantify the difference in computed tomography (CT) artefacts after implementation of carbon-fiber (CF) and titanium implants. This study utilized a quantitative technique to compare pre- and postoperative CT artifacts produced by CF and titanium implants in patients with spinal oligometastatic disease (OMD) undergoing stereotactic ablative radiotherapy (SABR). A greater increase in Hounsfield units (HU) was observed in the group treated with titanium spine implants than in the group treated with CF spine implants. This relatively high postoperative HU-increase after insertion of titanium implants indicated CT artefacts, while the relatively low HU-increase of CF implants was not associated with artefacts. Therefore, we propose a CT artefact grading system based on postoperative HU-increase. This could be used in future studies to assess if less CT artefacts due to treatment with CF implants lead to time savings during radiotherapy treatment planning and, potentially, better tumoricidal effects and decreased adverse effects if particle therapy would be administered.

## Data Availability

Raw data are available with the corresponding author and will be provided upon a written request.
